# Biomarkers in Emergency Medicine

**DOI:** 10.1155/2018/4597489

**Published:** 2018-03-20

**Authors:** Patrizia Cardelli, Mina Hur, Salvatore Di Somma

**Affiliations:** ^1^Sapienza University of Rome, Rome, Italy; ^2^Konkuk University, Seoul, Republic of Korea; ^3^Facoltà di Medicina e Psicologia, Roma, Italy

Researchers navigate the ocean of biomarkers searching for proper targets and optimal utilization of them. Emergency medicine builds up the front line to maximize the utility of clinically validated biomarkers and is the cutting edge field to test the applicability of promising biomarkers emerging from thorough translational researches. The role of biomarkers in clinical decision making would be of greater significance for identification, risk stratification, monitoring, and prognostication of the patients in the critical- and acute-care settings. No doubt basic research to explore novel biomarkers in relation to the pathogenesis is as important as its clinical counterpart. This special issue includes five selected research papers that cover a variety of biomarker- and disease-related topics.

The paper by N. Shi et al. demonstrated that the plasma microRNA-127 (miR-127) level was significantly downregulated in acute pancreatitis (AP) patients with respiratory failure compared with the healthy volunteers and those without respiratory failure. It was the first study that integrated miR-127 and the inflammatory injuries of the pancreas and the lung. miR-127 might serve as a potential marker for the identification of AP with lung injury.

The paper by L.-J. Chen et al. explored the association between plasma miRNA-24-3p (miR-24) expression and coagulation factor X (FX) and XII (FXII) levels in major trauma and trauma-induced coagulopathy (TIC) patients. In their study, miR-24 was overexpressed in major trauma and TIC patients, and miR-24 expression correlated with FX level negatively, suggesting the possibility that miR-24 might inhibit the synthesis of FX during TIC.

P. Lochner et al. measured osteopontin (OPN: biomarker for inflammation) and neurofilament heavy chain (NfH: biomarker for axonal injury) in patients with acute optic neuritis and sex- and age-matched healthy controls. They demonstrated that OPN and NfH are elevated in these patients, supporting the presence of underlying inflammation and axonal injury as well as the prognostic utility of examining biomarkers in optic neuritis.

The paper by B. Morawiec et al. addressed the prognostic value of copeptin in patients admitted with chest pain and suspected acute coronary syndrome (ACS). They concluded that copeptin appears to be an independent predictor of long-term mortality in patients with suspected ACS. Copeptin may be also considered as an early marker for identifying high-risk patients who would develop heart failure.

The last paper by C. Spiekermann et al. explored S100 proteins A8 and A9 (S100A8/A9) as a promising diagnostic marker for peritonsillar abscess (PTA). Using a combination of S100A8/A9 levels and characteristic symptoms of PTA, they also developed a PTA score as an objective and appropriate tool to differentiate between peritonsillitis and PTA.

We hope that this special issue would bridge basic research and clinical practice on biomarkers, facilitate the scientific development in this research field, and eventually contribute to the unrivaled value of disease markers.

*Patrizia Cardelli* *Mina Hur* *Salvatore Di Somma*

